# Pontine autosomal dominant microangiopathy with leukoencephalopathy: *Col4A1* gene variants in the original family and sporadic stroke

**DOI:** 10.1007/s00415-023-11590-9

**Published:** 2023-02-14

**Authors:** Jessica Roos, Stefanie Müller, Anne Giese, Silke Appenzeller, Erich Bernd Ringelstein, Jens Fiehler, Klaus Berger, Arndt Rolfs, Christian Hagel, Gregor Kuhlenbäumer

**Affiliations:** 1 Department of Neurology, University Hospital Schleswig-Holstein, Kiel, Arnold-Heller Str. 3, D24105 Kiel, Germany; 2 Institute of Health Informatics, University College London, London, UK; 3 Department of Neurology, University Medical Center, Hamburg-Eppendorf, Germany; 4 Comprehensive Cancer Center Mainfranken, University Hospital, Würzburg, Germany; 5 Medical Faculty, University of Münster, Münster, Germany; 6 Department of Diagnostic and Interventional Neuroradiology, University Medical Center, Hamburg-Eppendorf, Germany; 7 Institute of Epidemiology and Social Medicine, University of Münster, Münster, Germany; 8 Arcensus Gmbh, Rostock, Germany; 9 Institute of Neuropathology, University Medical Center, Hamburg-Eppendorf, Germany

**Keywords:** Pontine autosomal dominant microangiopathy with leukoencephalopathy, PADMAL, Autosomal dominant stroke, Sporadic stroke, *COL4A1*, Cerebral microangiopathy, Monogenic

## Abstract

**Background:**

(1) Description of clinical and cranial MRI features in the original Pontine Autosomal Dominant Microangiopathy with Leukoencephalopathy (PADMAL) family and correlation with the segregation analysis of the causative collagen 4A1 gene (*COL4A1*) variant. (2) Sequence analysis of the *COL4A1* miRNA-binding site containing the causative variant in two independent cross-sectional samples of sporadic stroke patients.

**Patients and methods:**

Sanger sequencing of the *COL4A1* miRNA-binding site in the PADMAL family and 874 sporadic stroke patients.

**Results:**

PADMAL shows adult-onset usually between 30 and 50 years of age with initial brainstem-related symptoms most commonly dysarthria, with progression to dementia and tetraparesis. Radiologically pontine lacunes are followed by supratentorial white matter involvement. Radiological onset may precede clinical symptoms. We found no variants in the *COL4A1* miRNA-binding site of sporadic stroke patients.

**Conclusion:**

Our results allow an early diagnosis of PADMAL based on cranial MRI, clinical signs, and confirmatory sequencing of the *COL4A1* miRNA-29-binding site. *COL4A1* miRNA-29-binding site variants do not contribute to a sizeable proportion of sporadic stroke.

**Supplementary Information:**

The online version contains supplementary material available at 10.1007/s00415-023-11590-9.

## Introduction

Sporadic cerebral microangiopathies are common and a leading cause of stroke and dementia [1]. Monogenic cerebral microangiopathies are comparatively rare but provide important models for the pathomechanisms underlying their sporadic counterparts [2]. Pontine Autosomal Dominant Microangiopathy with Leukoencephalopathy (PADMAL) is an autosomal dominant cerebral microangiopathy first described in a German family by Colmant and Hagel [3, 4]. PADMAL is an extremely rare disease. Only 11 families have been published and it is not possible to estimate the incidence or prevalence of the disease [5–9]. PADMAL belongs to the group of autosomal dominant cerebrovascular diseases caused by type IV collagen variants [10, 11]. Type IV collagens are the main constituent of basement membranes and form mesh-like structures with multiple functions including mechanic strength [12]. COL4A1/A2 proteins include a triple helix containing the classic Gly-Xaa-Yaa repeat amino acid sequence [12]. Missense variants affecting these glycine residues may lead to a failure to form proper triple helices [12]. Protein truncating variants and duplications may also cause cerebrovascular disease, presumably by reduced expression or overexpression of type IV collagens [11, 13–15]. Clinically, *COL4A1/A2* mutations cause a wide variety of phenotypes ranging from fetal death, porencephaly, and intracerebral hemorrhages to cerebral microangiopathy, cervical artery dissection, and some individuals even remain asymptomatic [10, 16, 17]. Extra-neurological features, such as renal involvement, cardiac involvement, and ocular disease, occur in a subset of variants [18]. PADMAL is caused by variants outside the protein-coding part of *COL4A1*, affecting a micro-RNA (miRNA-29)-binding site in the 3’-untranslated region (3’-UTR) [5–9]. These variants lead to a loss of transcription repression by miRNA-29 and consequently to collagen 4A1 overexpression [8]. These findings show that both, overexpression and under-expression of type IV collagens might lead to cerebrovascular disease and suggest that the expression of type IV collagens needs to be tightly regulated to maintain vascular integrity.

Interestingly, genetic variants in *COL4A1/A2* are also associated with sporadic cerebral microangiopathies [19–23]. However, no clear-cut relationship with *COL4A1*/*A2* mRNA expression has yet been demonstrated [23].

### Aim

Here, we (1) investigate the co-segregation of the causative *COL4A1* variant in the original PADMAL family complementing published clinical and radiological [4, 24] as well as pathological data [3, 4, 25] of this family and, and describe the clinical and radiological presentation and progression of PADMAL using this family and literature data (2) report a sequence analysis of the mutated miRNA-29-binding site in two German samples of patients with sporadic cerebral microangiopathies as well as other types of ischemic stroke.

## Patients and methods

### Patients, standard protocol approvals, registrations, and patient consent

All individuals of the original PADMAL family were examined in the years 2007–2014. Ages in this publication are ages at examination. Typically, we performed a full history and physical examination, a cranial Magnetic Resonance Imaging (MRI) scan, and obtained an EDTA-anticoagulated blood sample. We did not perform a cranial MRI in married-in family members. Technical details and results of the MRI investigations have been previously published [24]. For some bedridden individuals, clinical cranial MRI or computed tomography (CT) scans were obtained with their or next of kin’s consent. To preserve anonymity, we include no individual patient reports and present the pedigree without age and sex. All procedures performed in studies involving human participants followed the ethical standards of the institutional and/or national research committee and the 1964 Helsinki declaration and its later amendments or comparable ethical standards. All individuals gave written informed consent and institutional review board approval was obtained from the ethical advisory boards of the Universities of Hamburg and Kiel (Hamburg M276/06, Kiel B226/08).

The Muenster stroke sample included 225 patients with sporadic microangiopathic stroke according to the TOAST criteria. The Muenster stroke sample has previously been described in detail [26, 27]. We recruited in a completely anonymized fashion consecutive consenting ischemic stroke patients from hospitals in northwest Germany through the regional Westphalian Stroke Register and from the University Hospital of Greifswald in northeast Germany. The treating physicians used standardized patient assessment forms to collect sociodemographic and disease-related data as well as blood samples. For the whole study, we included all available patients with completed ischemic stroke, proved by computed tomography (CT) or magnetic resonance imaging (MRI) and classified as: large artery atherosclerosis (TOAST 1, *n* = 515), cardioembolism (TOAST 2, *n* = 411), or small vessel occlusion (TOAST 3, *n* = 255). We excluded patients who had experienced a transient ischemic attack (TIA) or hemorrhagic stroke, as well as patients falling into other TOAST categories, to maximize etiologic homogeneity. For this study, we analyzed all patients with small vessel occlusion (TOAST3) for whom DNA was still available (225 out of 255). All patients gave written informed consent and institutional review board approval was obtained from the ethical advisory board of Muenster and Kiel University (Muenster 00/113stö, Kiel D552/20).

The Rostock sample was ascertained in the context of the stroke in young Fabry patients (SIFAP) study and has been described in detail [28]. The whole Rostock Stroke Sample recruited initially, 5111 patients, 88 withdrew consent, including 1 patient with Fabry disease. Thus, 5023 patients aged between 18 and 55 years and presenting with an acute cerebrovascular event (CVE) of any cause within the last 3 months before recruitment were included in the study. Seventy-eight percent of all patients had been enrolled in the study within 10 days after the qualifying stroke event. The diagnosis of stroke had to be verified by brain MRI analysis (82%), or in case of negative or missing MRI, the clinical diagnosis of CVE had to be confirmed by a qualified stroke neurologist, who had at least 5 years of experience in treating stroke (18%). Patients not meeting these criteria had to be excluded from the study. All patients underwent thorough evaluation including brain and vascular imaging, and extensive laboratory testing according to the local laboratory routine (hemogram, blood fat, glucose, HbA1C, liver transaminases, creatinine, electrolytes, total albumin in serum, C reactive protein, antinuclear antibodies, anti-neutrophil cytoplasmic antibodies, rheumatoid factor, Factor V mutation, prothrombin mutation, antiphospholipid antibodies), Fabry disease diagnostics, cardiac ultrasound examination, and ECG. Data on comorbidities and vascular risk factors were collected in a standardized pre-specified case report form from medical records and self-reported by the patient.14 In addition, centers provided vascular and cardiac imaging, as well as stroke-associated comorbidities like depression, pain, or headache. For this study, we selected the samples of patients fulfilling the following criteria: (1) only patients who tested negative for Fabry disease and CADASIL, (2) only patients who consented to further genetic studies and the sharing of samples as well as data with collaborators, (3) from this subsample, patients with MRI signs of cerebral microangiopathy, either lacunar stroke or leukoaraiosis Fazekas grad 2/3 and/or vertebrobasilar involvement/stroke. All patients gave written informed consent and institutional review board approval was obtained from the ethical advisory board of Rostock and Kiel University (Rostock II PV 03/2006, Kiel D552/20).

### Sequence analysis of the *COL4A1* miRNA-29-binding site

Genomic DNA was isolated from EDTA-anticoagulated blood samples using standard procedures in all but deceased patients from the PADMAL family in whom DNA was obtained from paraffin-embedded brain sections. The miRNA-binding site and surrounding DNA sequence were Polymerase Chain Reaction (PCR) amplified for 35 cycles using the oligonucleotide primers COL4A1_UF1 (5′-TACGCCGTCCACCTTGAA-3′) and COL4A1_UR1 (5′-AGGTCAATGAAGCAGGGTGT-3′) employing standard procedures. Sanger sequencing was performed using BigDye Terminator chemistry (Applied Biosystems, MA, USA) on an Applied Biosystems 3730xl DNA analyzer (Applied Biosystems, MA, USA). Sequence electropherograms were assembled and compared to the human genome reference sequence (build GRCh38/hg38) using the SeqMan module of the Lasergene software (DNAstar, WI, USA).

### Power analysis

We tested for the statistical power to detect at least one individual with a variant in the analyzed region of the *COL4A1* gene versus none at a *p* value threshold of *p* < 0.05. We used RStudio (Version 2022.07.1 Build 554) with the package “pwr” and the function “pwr.p.test” with mutation frequencies between 0 and 0.5 percent and a sample size of 874 individuals.

## Results

### Segregation analysis in the PADMAL family

**Fig. 1:**
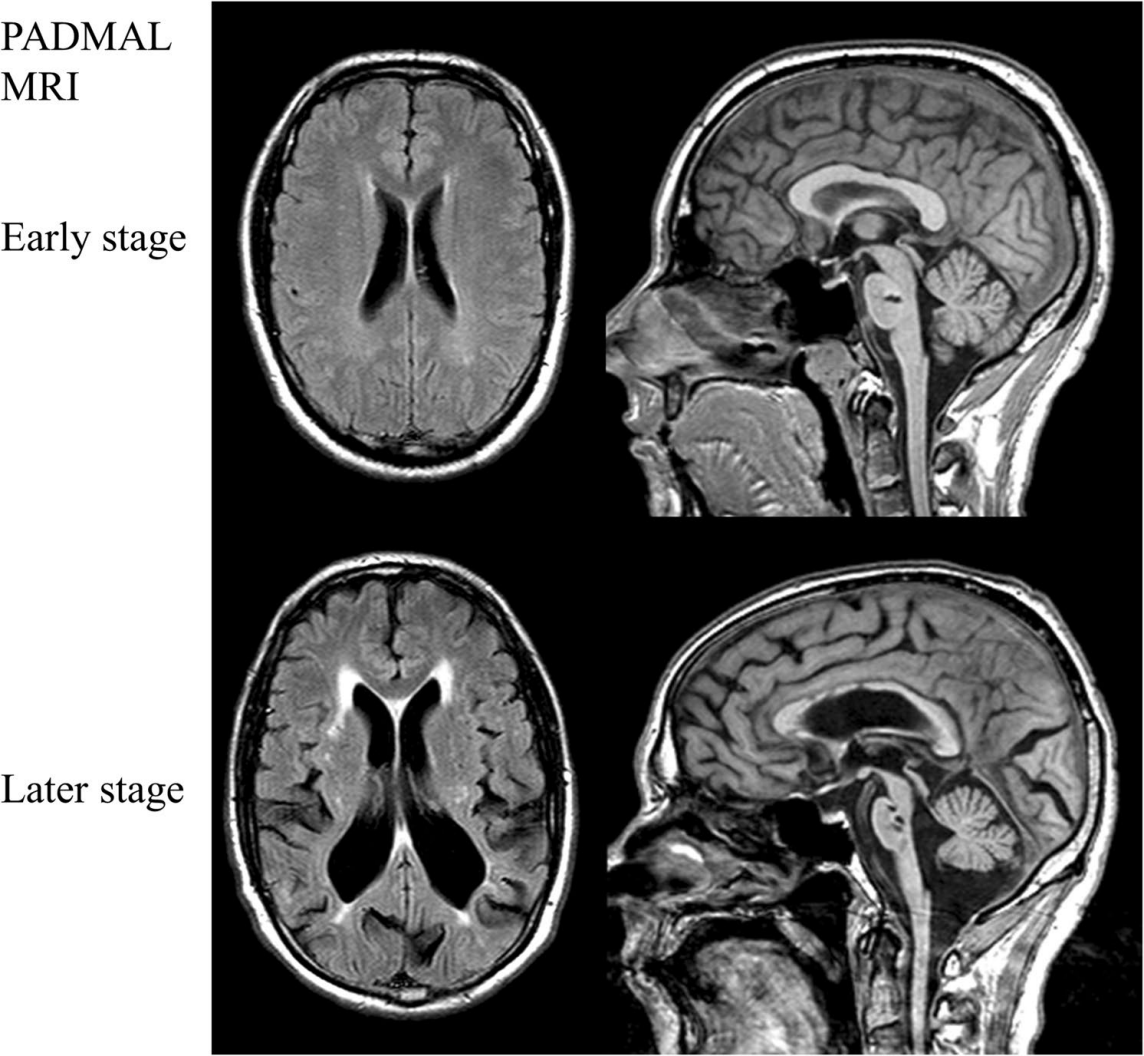
MRI features of PADMAL. T2-weighted cranial MRI scans of an early and a later stage PADMAL patient demonstrating pontine and supratentorial involvement

**Fig. 2:**
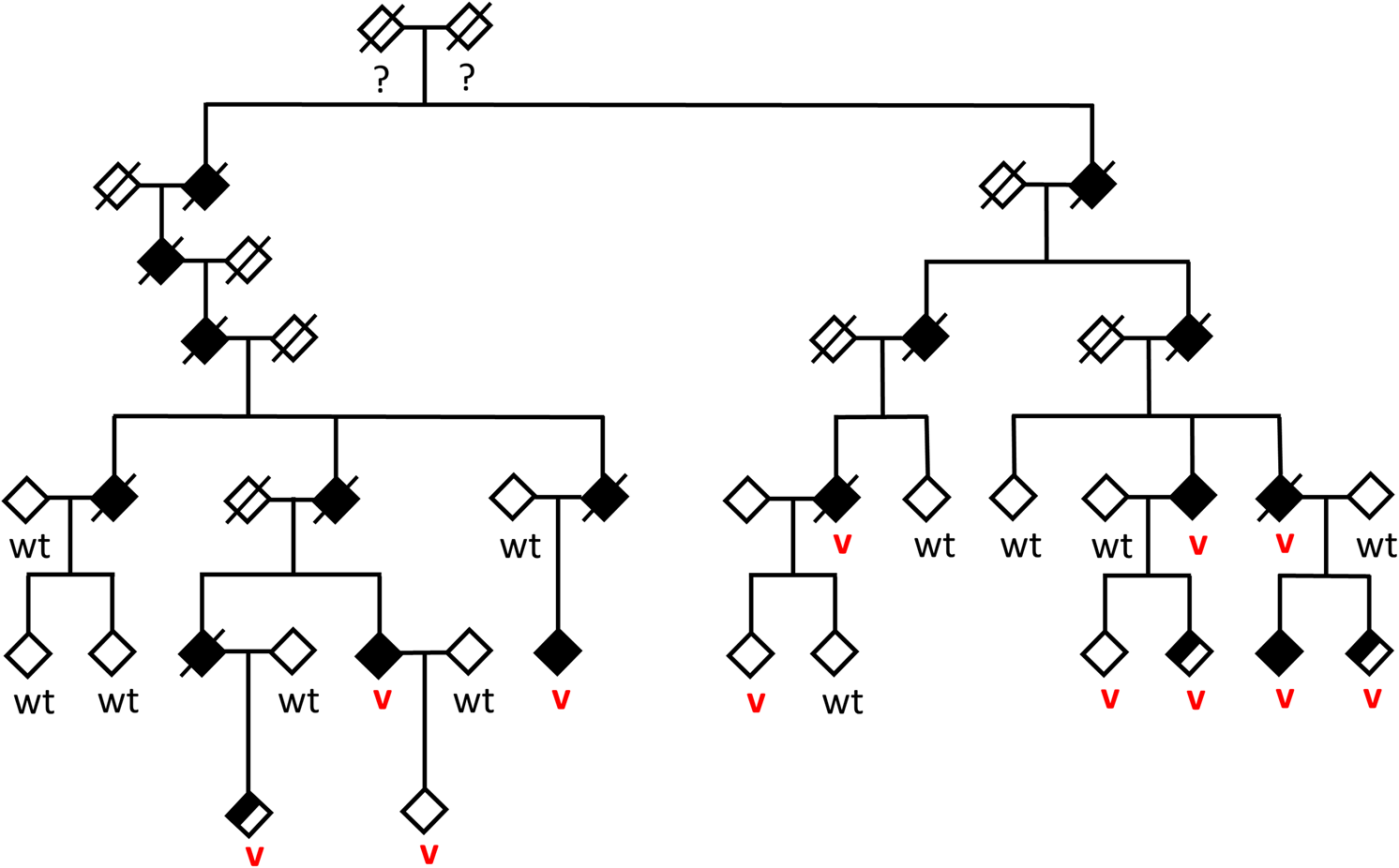
Anonymized pedigree of the family with PADMAL. Empty symbols show clinically and radiologically unaffected individuals. Half-filled symbols show radiologically affected but clinically unaffected individuals. Filled symbols show radiologically and clinically affected individuals. Slashed symbols show deceased individuals. Red “v” stands for heterozygous *COL4A1*, NM_001845.6:c.*31G > T carriers and wt for individuals who are homozygous wildtype. Sex is omitted from the pedigree

**Fig. 3:**
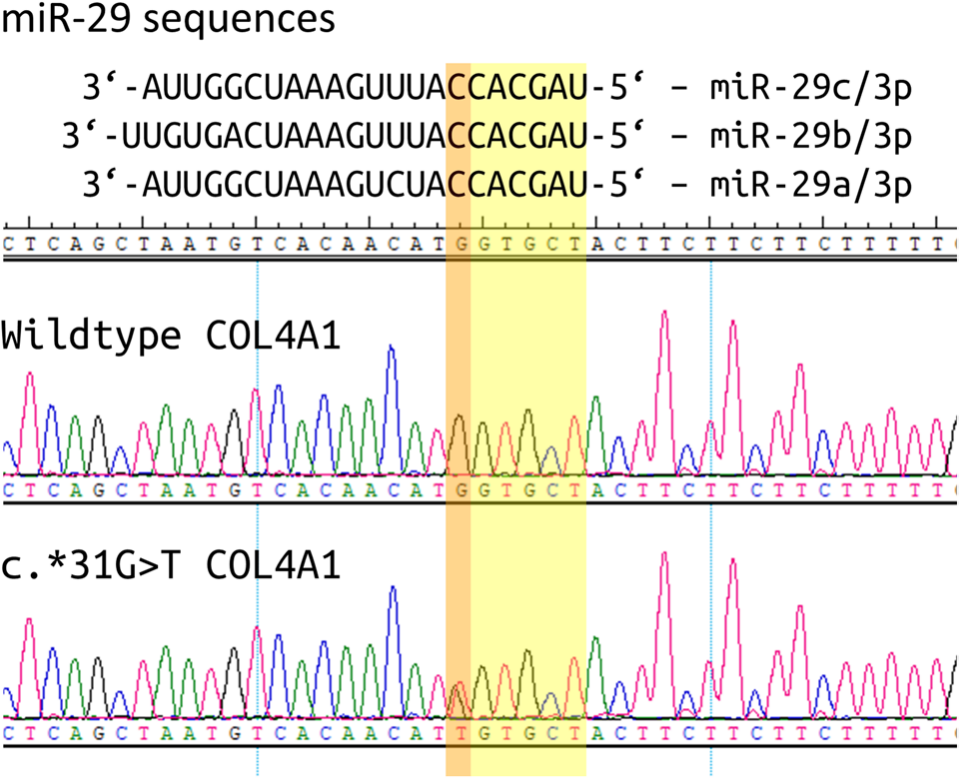
*COL4A1*, NM_001845.6:c.*31G > T variant causing PADMAL in this family. The upper panel shows miRNA-29 variants within the binding site. The middle panel shows the *COL4A1* wildtype sequence and the lower panel the *COL4A1*, NM_001845.6:c.*31G > T variant in a patient from the family with PADMAL

We defined radiological affection status based on the presence of at least one lacune in the pons because pontine lacunes were always present in MRIs demonstrating supratentorial white matter lesions (WML) while isolated pontine involvement—even in the absence of clinical symptoms and signs—was present in some individuals (Fig. 1). Age at radiological onset was defined as the age at the first MRI showing evidence of PADMAL. We based clinical affection status on a history of stroke, a history of symptoms suggesting a stroke, and clinical signs suggesting a past stroke in radiologically affected individuals. The anonymized pedigree (Fig. 2) shows that genotypes were available for 17 family members excluding married-in individuals of whom 6 were clinically and radiologically affected, and 3 were only radiologically affected. Three individuals were clinically and radiologically unaffected but carried the causative *COL4A1* variant and were therefore defined as at risk. Figure 3 shows the causative *COL4A1* variant in the sequence context of the miRNA-29-binding site with the cognate miRNAs. Table 1 summarizes the main clinical and radiological characteristics of the affected and the at-risk individuals at the time of examination. Despite the small number of patients, it becomes apparent that the radiological onset is around a decade earlier than the clinical onset. However, the radiological onset can not be estimated reliably because it is determined by the time of the first MRI. Note that the radiological onset in the individuals who are also affected clinically seems to be later than in individuals with radiological signs but no clinical symptoms. This is an artifact caused by the fact that the first MRI in the clinically affected individuals was performed at the clinical onset. In all but one clinically affected individual, the first symptom (dysarthria, vertigo, ataxia) was most likely related to a brainstem stroke. The death occurred in the 6th to 8th decade.

**Table 1:** Summary of radiological and clinical characteristics of individuals carrying the causative *COL4A1* variant

	Affected: radiologically + clinically	Affected: radiologically	At-risk
Age at examination mean (SD)	58 (10)	36.0 (1)	38 (6)
Age at radiological onset mean (SD)	52 (8)	36 (1)	Not affected
Age at clinical onset mean (SD)	52 (10)	Not affected	Not affected
First symptoms	Dysarthria (3) vertigo (2)	None	None
	Ataxia (2)		
	Hemiparesis (1)		
Further symptoms	Tetraparesis (2) unknown (4)	None	None
	Mutism (2) dementia (3)		
Age at death mean (SD)	67 (4)	NA	NA

*SD* standard deviation

### Sequence analysis of the *COL4A1* miRNA-29-binding site in two German sporadic stroke samples

In the Muenster stroke sample, genomic DNA was still available for 225 of 255 patients with microangiopathic stroke described in a previous publication [26]. Table 2 summarizes the demographic and clinical data for the Muenster and Rostock sporadic stroke samples. The Muenster sample represents a typical cross-section of patients with a microangiopathic stroke. The Rostock sample (649 patients) consists of young-onset stroke patients with preferential brainstem and cerebellar localization but with diverse etiologies [28]. Therefore, the age at stroke is higher (mean 68 vs. 44 years) and more variable (standard deviation of 13 vs. 8 years) in the Muenster sample compared to the Rostock sample. The Muenster sample is sex balanced while the Rostock sample shows a male preponderance (50% vs. 66% male). Detailed information on the lesion distribution is present for the Rostock but not for the Muenster sample. Power analysis (Supplemental Fig. 1) showed a power of 80% to detect a variant in the *COL4A1* miRNA-29-binding site at a frequency of 0.18% for our sample of 874 patients.

**Table 2:** Demographic and clinical characteristics of the Muenster and Rostock sporadic stroke samples

	Muenster sample	Rostock sample
Number of patients	225	649
Sex: female *n*/%, male *n*/%	113 (50), 112 (50)	220 (34), 429 (66)
Age at stroke: mean (SD)	68 (13)	44 (8)
Cerebral macroangiopathy: *n* (%)	0	103 (16)
Cardioembolic: *n* (%)	0	106 (17)
Cerebral microangiopathy: *n* (%)	225 (100)	66 (11)
Other defined etiology: *n* (%)	0	165 (26)
Undefined etiology: *n* (%)	0	190 (30)
Supratentorial cortical-subcortical: *n* (%)	NA	109 (17)
Supratentorial white matter: *n* (%)	NA	53 (8)
Basal ganglia: *n* (%)	NA	30 (5)
Thalamus: *n* (%)	NA	50 (8)
Brainstem: *n* (%)	NA	301 (46)
Cerebellum: *n* (%)	NA	411 (63)

*n* number of individuals, (%)—number of individuals as percent of the total, *SD* standard deviation

Sequence analysis in two independent German sporadic stroke samples did not identify a single variant in the core *COL4A1* miRNA-29-binding site (5′-GGTGCT-3′) in any of the 874 sequenced patients with sporadic stroke.

## Discussion

This manuscript serves two purposes. First, it shows the co-segregation of a *COL4A1* 3’UTR variant with the disease in the family for which the name Pontine Autosomal Dominant Microangiopathy with Leukoencephalopathy (PADMAL) was coined. This completes our previous reports on clinical features, MRI findings, and pathology and underpins the causality of the detected variant [3, 4, 8, 24]. Taken together and adding the results of other publications, a delineation of the sequence of clinical and radiological progression of the disease is possible. The first radiological signs are pontine lacunes most likely appearing in the 4th to 5th decade in some individuals while others present with normal MRIs in this age range. Whether the latter individuals remain asymptomatic due to reduced penetrance can not be answered yet. There is uncertainty in the age at radiological onset because detection depends on a cranial MRI performed in a clinically healthy individual which is usually not performed outside a study context. Therefore, radiological onset might be somewhat earlier than in this study. The clinical onset, mostly in the form of brainstem-related neurological symptoms is in the 4th to 6th decade. The disease then progresses fairly rapidly and leads to death in the 6th to 8th decade. The first MRI signs are brainstem lacunes, followed by supratentorial white matter lesions and atrophy of pons and medulla oblongata. Several reports describing families or single cases suffering from PADMAL have been published [5, 7–9, 29, 30]. For none of these families, extensive MRI studies are available which could be compared in detail to our study [24]. The causative variants vary slightly between studies but all affect the *COL4A1* miRNA-29-binding site. Nevertheless, data in these studies allow the conclusion that the disease started in most cases with brainstem symptoms, namely with dysarthria. There is also some variation in the age of onset and the age at death between studies but adult-onset (mostly 4th to 5th decade) and disease duration between 10 and 20 years were found in all patients. Compared to disease caused by variants in the coding regions of *COL4A1* and *COL4A2*, PADMAL has a later age at onset and does not lead to cerebral hemorrhages, or porencephaly. The second purpose of this manuscript is to answer the question of whether some sporadic stroke patients also show a variant in the *COL4A1* miRNA-29-binding site, we sequenced 874 German stroke patients, of whom 291 suffered from a microangiopathic stroke but did not find a single variant in the *COL4A1* miRNA-29-binding site. Our power analysis shows that it is unlikely that variants in the *COL4A1* miRNA-29-binding site are present at a frequency above ~ 0.2%. Of note, the *COL4A1* miRNA-29-binding site is extremely conserved across species, none of the mammals, birds, and bony fish available for analysis in the UCSC human genome browser (Supplemental Fig. 2 and https://genome.ucsc.edu) show a single base change. Further, no variant in the 6-base-binding motif is found in the approximately 117.007 human sequences covering this motif in the gnomAD database (https://gnomad.broadinstitute.org, GRCh38/hg38, NC_000013.11:g.110.150.327–110.150.332) and no single-nucleotide polymorphism has been reported up to dbSNP version 153 (https://www.ncbi.nlm.gov/snp/). Targeted sequencing of genes causing monogenic disease has in some cases revealed a higher frequency of pathogenic variants in sporadic patients or a contribution of less pathogenic variants to sporadic diseases. An example of the former is Fabry disease which is a moderately common cause of young-onset stroke [28]. An example of the latter is the contribution of common heterozygous variants in the gene causing Gaucher disease in the pathogenesis of Parkinson’s disease [31, 32].

Our study has several limitations. The first is the small number of family members available for clinical and radiological analysis in the PADMAL family. More family members would allow a more detailed description of the phenotype. However, compared to other studies of PADMAL, we have analyzed one of the largest families. The second is the size and composition of the samples with sporadic stroke. All patients in the Muenster sample had a diagnosis of cerebral microangiopathy but the lesion distribution is unknown. Therefore, we do not know in whom the lesion pattern was similar to PADMAL. For the Rostock sample, this data was available. The much younger age at stroke onset increases the likelihood of a monogenic cause. We can not exclude that variants in the *COL4A1* miRNA-29-binding site contribute to the pathogenesis of sporadic stroke at low frequencies below ~ 0.2% or to specific stroke subtypes not sufficiently represented in our samples. A third limitation might be the lack of functional data. However, these data have already been provided by other publications [6, 8, 25].

## Conclusion

PADMAL is an extremely rare autosomal dominant cerebral microangiopathy caused by variants in the *COL4A1* miRNA-29-binding site. Our clinical description in conjunction with other publications delineates an autosomal dominant inheritance pattern, adult-onset (mostly 4th to 5th decade), initial brainstem-related symptoms (most commonly dysarthria), progression to tetraparesis and dementia and pontine lacunes and atrophy followed by supratentorial white matter lesions as the main clinical and radiological characteristics which should suffice to raise the suspicion of PADMAL. Variants in the *COL4A1* miRNA-29-binding site are not involved in the pathogenesis of a sizeable proportion of sporadic stroke.

## Supplementary Information

Below is the link to the electronic supplementary material.

Supplementary file1 (DOCX 625 KB)

## Data Availability

All data pertaining to *COL4A1* miRNA-29-binding site sequencing are contained in the article itself.
