# Supplementary material for: Pontine autosomal dominant microangiopathy with leukoencephalopathy: Col4A1 gene variants in the original family and sporadic stroke
Source: J Neurol. 2023 Feb 14;270(5):2631–9. doi: 10.1007/s00415-023-11590-9 (PMC10130117; doi:10.1007/s00415-023-11590-9)
Supplement: Supplementary file 1 — Supplementary file1 (DOCX 625 KB) [file 415_2023_11590_MOESM1_ESM.docx]

Supplemental data to: Roos et al. Pontine Autosomal Dominant Microangiopathy with Leukoencephalopathy: *Col4A1* gene variants in the original family and sporadic stroke

Supplemental figure 1: Power analysis of the COL4A1 miRNA binding site sequencing in two stroke samples


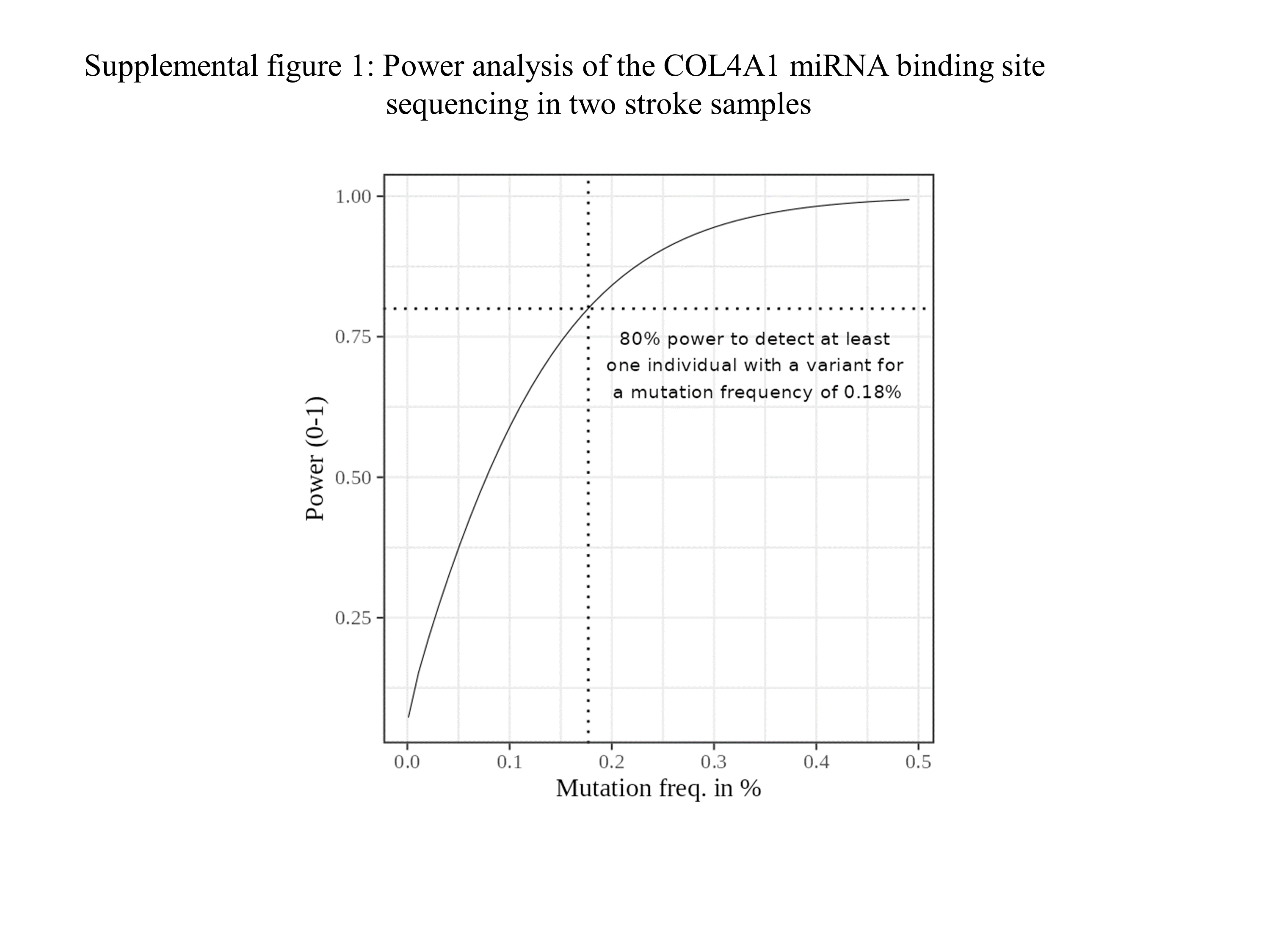


Supplemental figure 1: Statistical power (for p = 0.05) was calculated using the “pwr” package in R as detailed in the “Patients and Methods” section. Power is displayed as a function of the mutation frequency in percent. The crossing point of the dotted lines indicates 80% power for a mutation frequency of 0.18%.

Supplemental figure 2: Conservation of the *COL4A1* miRNA binding site (genomic)


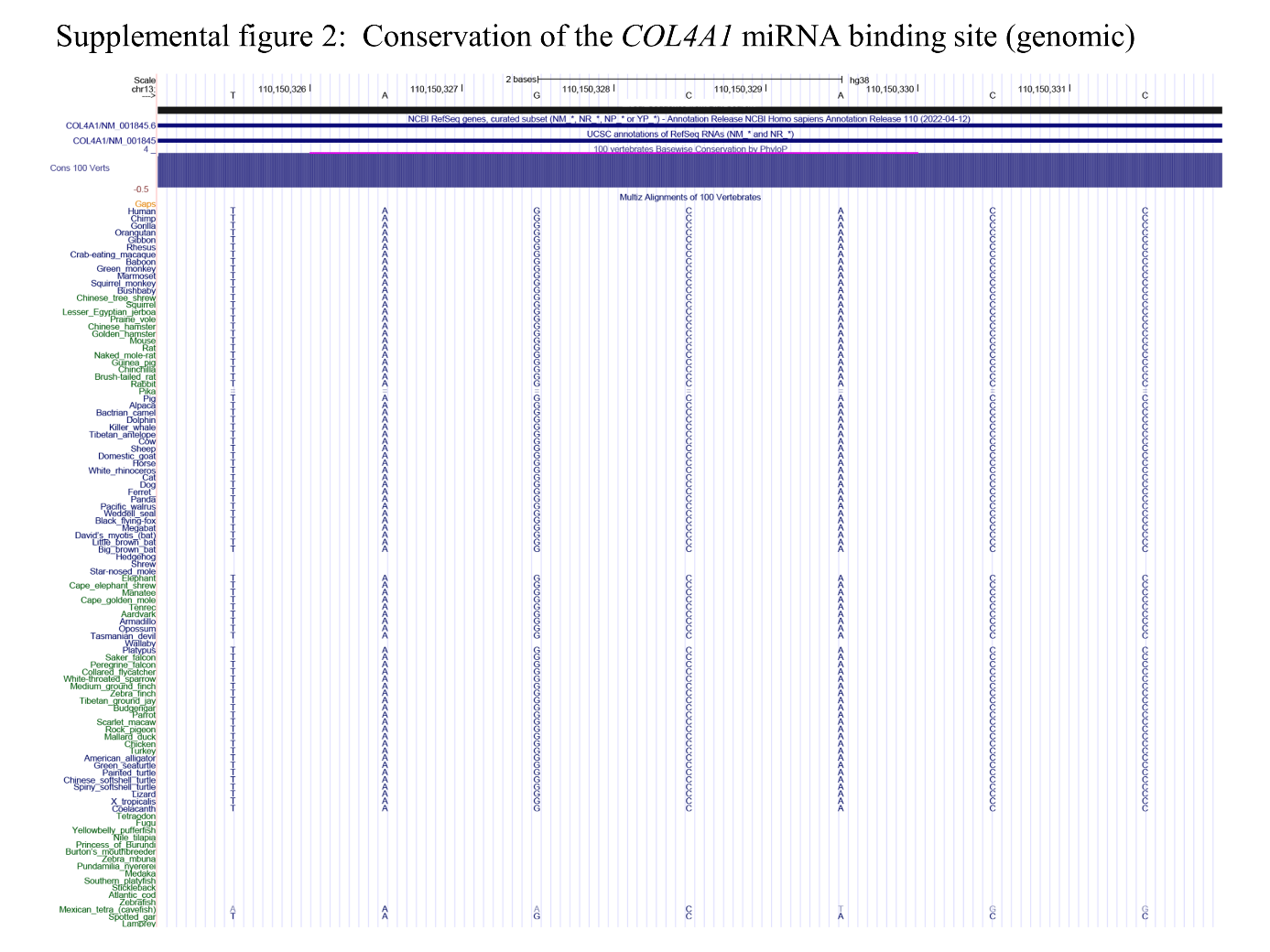


Supplemental figure 2: The conservation data are derived from the UCSC human genome browser (<https://genome.ucsc.edu/>). The sequence is displayed on the genomic forward strand while *COL4A1* is encoded on the reverse strand.
